# Three Cases of Severe ME/CFS in Adults

**DOI:** 10.3390/healthcare9020215

**Published:** 2021-02-16

**Authors:** Leah R. Williams, Carol Isaacson-Barash

**Affiliations:** Massachusetts ME/CFS & FM Association, Quincy, MA 02269, USA; cibarash@helixhealthadvisors.com

**Keywords:** myalgic encephalomyelitis (ME), chronic fatigue syndrome (CFS), severe ME/CFS, very severe ME/CFS, post-exertional malaise (PEM)

## Abstract

Myalgic encephalomyelitis/chronic fatigue syndrome (ME/CFS) is a complex, only partially understood multi-system disease whose onset and severity vary widely. Symptoms include overwhelming fatigue, post-exertional malaise, sleep disruptions, gastrointestinal issues, headaches, orthostatic intolerance, cognitive impairment, etc. ME/CFS is a physiological disease with an onset often triggered by a viral or bacterial infection, and sometimes by toxins. Some patients have a mild case and are able to function nearly on a par with healthy individuals, while others are moderately ill and still others are severely, or even, very severely ill. The cohort of moderately to very severely ill is often housebound or bedbound, has lost employment or career, and has engaged in a long, and often futile, search for treatment and relief. Here, we present three case studies, one each of a moderately ill, a severely ill, and a very severely ill person, to demonstrate the complexity of the disease, the suffering of these patients, and what health care providers can do to help.

## 1. Introduction

Myalgic encephalomyelitis/chronic fatigue syndrome (ME/CFS) is a complex multi-system disease that impacts the immune, endocrine, neurological, and energy production pathways in the body. Current diagnostic criteria include the hallmark symptom of post-exertional malaise (PEM), meaning a prolonged exacerbation of symptoms following mental or physical exertion, and fatigue, unrefreshing sleep, cognitive impairment, and/or orthostatic intolerance that has persisted for more than six months and that reduce or impair the ability to engage in pre-illness activities [1,2]. Defined as a neurological disease by the World Health Organization since 1969 [3], ME/CFS has been mistakenly characterized as a mental disorder by some groups, leading to stigmatization and a lack of appropriate care [4]. There are no biomarkers or validated diagnostic tests and no US Food and Drug Administration (FDA)-approved treatments.

ME/CFS is not a rare disease. There are at least an estimated 1.5 million people with ME/CFS (pwME) in the US [1,5,6]. Roughly three times as many women as men are affected [1]. Although the etiology is unclear, many cases follow a viral infection [7]. In a prospective study in Australia, 11% of patients with acute infections of Epstein–Barr virus (EBV), Q-fever (*Coxiella burnetii*), or Ross River virus (an RNA alphavirus) met the criteria for ME/CFS at six months [8]. Studies of the long-term sequelae of severe acute respiratory syndrome (SARS) found that 27% met the diagnostic criteria for ME/CFS after four years [9]. The current SARS-CoV-2 pandemic is expected to lead to a large increase in the number of pwME [10,11,12,13,14].

The frequency and severity of ME/CFS symptoms can vary from day-to-day and week-to-week, and symptoms can range from mild to severe. Carruthers et al. 2011 [15] defined a severity scale for ME/CFS of mild (at least a 50% reduction in pre-illness activity level), moderate (mostly housebound), severe (mostly bedridden), and very severe (completely bedridden and requiring assistance with basic functions). Studies of disability in pwME have estimated that 75% are housebound most of the time, 50% are unemployed, and 25% are bedbound most of the time [7,16,17]. Full recovery is rare (estimated at less than 5%), although some pwME experience remission of symptoms for extended periods, followed by relapse [7,18].

In this paper, we present three case studies of pwME, one moderately ill, one severely ill, and one very severely ill. Although children and adolescents get ME/CFS, in this paper, we focus on adults. Our goal is to describe the course of the disease and the extensive search for treatments undertaken by pwME and their health care providers, highlighting the complexity of ME/CFS and the suffering of patients. Several common themes emerge, including the difficulty of obtaining a diagnosis, the difficulty of finding supportive doctors, and the lack of treatments.

## 2. Case A

This 60-year-old white male was diagnosed with chronic EBV syndrome in 1986 by an internist. Symptoms leading to this diagnosis appeared following a 1983 martial arts injury and exacerbating spondylolysis at L5/S1. The injury was treated with a Boston Bucket back brace, which was worn for 11 months. During this time, the patient was cared for by a team of physiatrists, neurologists, and orthopedists. Six months into the treatment, the patient developed recurring bouts of debilitating fatigue, muscle pain, and painful bronchial and sinus discharge lasting for two to five days at roughly six-week intervals. The patient was seen by pulmonary, allergy, and infectious disease doctors who could find no explanation for the symptoms. The consensus among the care team was that the patient’s “... chronic complaint of fatigue and muscle ache may be secondary to lack of exercise, secondary to his low back injury.” The same team later diagnosed “chronic bronchitis with upper respiratory tract infection of uncertain etiology.” A year-long series of endocrine and immunologic testing revealed no abnormalities. The patient was referred for a psychiatric consult. The psychiatrist’s notes stated the following: “I believe this young man is malingering or has Munchausen syndrome.” At this point, the patient sought a second psychiatric opinion. After three visits, the patient was told “you need a good internist who can help figure this out.” This led to consultation with the internist and the previously noted diagnosis of chronic Epstein–Barr virus syndrome.

The patient was treated with low dose tricyclics and antibiotics. He also began acupuncture and chiropractic care and psychiatric counseling. Over several years, the medical diagnosis changed to chronic fatigue immune dysfunction syndrome (CFIDS) and then chronic fatigue syndrome (CFS). The patient became active in a local support group and participated in several research studies. With these supports, he continued to work full-time, including extensive travel, and he started a family, although episodic flare-ups of exhaustion and brain fog (or “crashes”) and recurring bouts of bronchitis/sinusitis resulted in frequent sick days and the need to work from home, and a three-month leave of absence.

Over the next 14 years, the frequency and severity of crashes diminished. By 1999, the limitation of the illness became a secondary factor in the patient’s daily life and he began to consider himself recovered. In 2012, he contracted pneumonia, and then, six months later, following removal of a tick, he was diagnosed with Lyme disease and anaplasmosis. He developed constant, disabling fatigue, cognitive impairment, severe post-exertional malaise, light-headedness, muscle pain, and gastrointestinal problems. The patient was bedridden for several weeks and then began an aggressive search for symptom relief, coordinated jointly by his internist and an ME/CFS specialist. He was misdiagnosed with sleep apnea in 2013, leading to the identification of deviated septum, concha bellosa, and turbinate hypertrophy, which were corrected by endoscopic sinus surgery in 2014. Surgery did not ameliorate symptoms. In a search for the cause of the sinus problems, he was evaluated for immune deficiencies by an ME/CFS specialist in 2014, revealing a mild deficiency in immunoglobulin G (IgG1). A further study that same year by an immunologist found an absence of 10 pneumococcal serotypes. He received a Pneumovax vaccination, which produced a significant decrease in the occurrence of both sinus and bronchial infections. Later in 2014, in pursuit of an explanation for the debilitating fatigue, he was evaluated by a geneticist who identified a heterozygous mutation in PSTPIP1 and recommended mitochondrial cofactor support as a way to decrease oxidative stress. A mitochondrial cocktail was prescribed. In consultation with the patient’s psychiatrist, Ritalin was added to the cocktail. After six months, this regimen resulted in improved energy levels with less dramatic highs and lows. In search of better indicators of mitochondrial dysfunction, the patient underwent a mitochondrial function test [19], which produced a mitochondrial score of 0.24 (24% energy availability at the cellular level) and revealed significant blocking of mitochondrial active sites and translocation proteins. Adjustments to the patient’s mitochondrial cocktail were recommended. However, these adjustments had no bearing on symptomology.

In 2014, the patient was also referred to a pulmonologist who suspected dysautonomia and administered an invasive cardiopulmonary exercise test (iCPET) [20]. This testing revealed pronounced deficiencies in the anaerobic threshold, ventricular refill, and oxygen uptake by muscles, resulting in the diagnoses of preload failure, mitochondrial myopathy, and disordered ventilatory control. Treatment with Mestinon (pyridostigmine bromide) and mild exercise with a recumbent bicycle helped the patient recover enough stamina to resume a modest work schedule. Endocrine, neurologic, and hematology assessments between 2017 and the present uncovered De Quervain’s thyroiditis, treated with Synthroid and resolved within 24 weeks; generalized autonomic failure and orthostatic hypertension, for which no effective treatments were identified; vestibular migraine, treated with some benefit by vitamin B2; and monoclonal gammopathy (MGUS), which is being monitored.

Presently, the patient is able to work approximately 16 hours a week and, while frequently resting at home, is no longer housebound. His treatment regimen includes monthly talk therapy, weekly shiatsu and chiropractic treatments, daily Qi Gong, supplemental oxygen as needed, and the following medicines and supplements: Ritalin, Mestinon, Glutathione, Co-Q10, Magnesium, vitamins B2, B12, C, and D, HSO probiotics and Enhance(r), a Chinese herbal antiviral.

This case demonstrates that aggressive pursuit of treatable symptoms can yield benefits for the patient. While there are no FDA-approved treatments for ME/CFS itself, several comorbid conditions were identified and were successfully treated. In addition, some symptoms, such as pain, sleep difficulties, and autonomic problems, can be addressed with medication. Alternative therapies and major lifestyle changes also contributed to improved quality of life. Finally, this case shows the heterogeneous nature of ME/CFS, with two distinct periods of disease separated by over a decade of good health, each period with different etiology and symptoms.

## 3. Case B

This case involves a 44-year-old white female who has had ME/CFS symptoms for almost 20 years, although she did not receive a diagnosis until 2014. Her symptoms started gradually in 2001 after blunt head trauma and a tick bite, with exhaustion being the main complaint. Consultation with a doctor in 2002 was unhelpful because routine bloodwork revealed nothing outside normal limits. This doctor told her she was the “epitome of good health.” Her symptoms of fatigue and post-exertional malaise increased in severity over the next few years. She consulted with internal medicine doctors, gynecologists, psychologists, and psychiatrists. In 2004, she was treated by a gynecologist who observed normal results on a blood workup and misdiagnosed her with depression. She was prescribed an antidepressant, which did not improve symptoms. Several chiropractors and acupuncturists tried adjustments, acupuncture, dietary changes, and traditional Chinese medicine (TCM) to improve her energy, none of which helped. Determined to heal herself, she became a certified yoga instructor in 2010, after which she participated in several advanced training workshops, which focused on improving energy in the body. She reports that the training and practice of the methods taught during training only led to decreases in her energy level.

In 2011, an endocrinologist determined that she was post-menopausal (at age 35) and prescribed hormone replacement therapy (HRT). Although HRT relieved night sweats and hot flashes, it did nothing to improve energy levels or reduce fatigue and PEM. Medical doctors continued to dismiss or misdiagnose her symptoms of overwhelming fatigue, PEM, cognitive dysfunction, unrefreshing sleep, light sensitivity, headaches, sore throat, and irritable bowel syndrome, all of which continued to worsen. From her own research, she realized that these symptoms aligned with the symptoms of ME/CFS. In 2014, a doctor of osteopathic medicine diagnosed her with ME/CFS after learning of her self-diagnosis and researching the disease himself. He recommended supplements, which did not improve her condition. Unable to find a local doctor with expertise in ME/CFS, she found a doctor who could treat her remotely. This doctor also recommended supplements, which did not help, and a Paleo Diet, which helped alleviate the irritable bowel syndrome but did not give relief from the fatigue or PEM.

By 2014, she was unable to continue performing the physical aspects of her job as a wellness coach and yoga instructor and instead enrolled in a master’s degree program for counseling psychology, taking one class per semester. At this point, she was mostly housebound. Two years later, she lost the ability to read and had depleted her savings paying rent and other bills. She was forced to drop out of graduate school and move back into her parents’ home. By then, she was so weak she spent more than 23 hours a day in bed. Her cognitive function was so poor that she was unable to even read a magazine article. She could not practice guitar, sing, or do art. She experienced such severe nausea that she sometimes could not eat. She was so sensitive to light and sound that she rarely left her darkened bedroom. She could not have visitors because talking was too taxing.

In 2017, she applied for Social Security Disability Insurance (SSDI). Her application was denied due to the inaccurate assessment in the denial letter that she was able to “be on (her) feet most of the day” and “lift up to 10 pounds frequently.” She appealed the denial and was again turned down, even with extensive documentation of her level of disability by her primary care physician. She requested a hearing by an administrative law judge. In the meantime, she underwent an invasive cardiopulmonary exercise test (iCPET) and was diagnosed by a pulmonologist with severe autonomic dysfunction in the form of preload failure [21]. She was also tested for and diagnosed with small fiber polyneuropathy by a neurologist [22]. These two diagnoses helped convince the administrative law judge to approve her SSDI application.

Treatment with Mestinon (pyridostigmine bromide) for preload failure and with midodrine for low blood pressure helped to slightly improve her functioning. In 2018, she started seeing an integrative medicine doctor who specializes in treating ME/CFS patients. He treated her with an antiviral (for trace findings of Epstein–Barr virus), an antibiotic (for trace findings of tick-borne illnesses), and vitamin B-12 injections. The first two treatments had no effect, while the B-12 seemed to help with getting out of bed and walking around. She currently spends 21 to 23 hours per day in bed and is able to listen to an audiobook or watch TV for up to two hours per day.

The increased availability of telemedicine in the US during the COVID-19 pandemic has dramatically improved the ability of this patient to access medical care. Previously, travel to and from a medical appointment, combined with waiting in a doctor’s office and the appointment itself, caused severe PEM that lasted for weeks. While virtual appointments still cause PEM, it only lasts for a few days. In addition, because the virtual appointments are more manageable, she has not had to reschedule as often.

This case demonstrates the harm that disbelieving health care providers inflict on patients with ME/CFS. Had this patient received an early diagnosis and education on managing her illness with pacing and self-care [2,23], she might not be as severely disabled today. In addition, this case shows the enormous losses suffered by the severely ill, including losing a career, not being able to live independently, and giving up the dream of having children. These losses are compounded by fear about a future when her parents are no longer available to be her caregivers.

## 4. Case C

This white female was 38 years old when she died in 2019 after years of suffering from very severe ME/CFS. She first developed symptoms of ME/CFS as a teenager, although she was not diagnosed for nearly 12 years. In 1999, at age 18, she had a serious case of EBV (mononucleosis) that kept her home from school for three months and required treatment with steroids for lymphadenopathy. Subsequently, she experienced variable episodes of fatigue and daytime sleepiness that gradually increased in severity. The corresponding increase in brain fog and decrease in energy made university level study difficult. Having been an athlete in high school, she tried to maintain an exercise regime for many years, despite worsening symptoms and PEM. Routine blood workups at this time, and at many times in the next 20 years, revealed nothing outside of normal limits.

As a junior in college, she was evaluated by a psychiatrist, who ruled out anxiety, depression, and somatization as sources for the fatigue. She also underwent a neuropsychological evaluation that ruled out any innate learning disability or attention deficit disorder. She was rated in the 99th percentile for intellectual ability, but only in the 39th percentile for reading rate, consistent with self-reported difficulty concentrating and processing information. Despite these challenges, she earned a BA in Biology and started a master’s degree program in Environmental Science.

In 2004, at age 23, she developed pain and weakness in her hands, arms, neck, and shoulders to the point where she could no longer type and was forced to take a one-and-a-half year leave of absence from graduate school. She was diagnosed with fibromyalgia and myofascial pain syndrome by her primary care physician. Treatments included physical therapy, sports massage, myofascial release therapy, and acupuncture; these provided some relief from the pain. In 2005 and 2006, she had two bouts of diverticulitis with micro-perforations, requiring hospitalization and IV antibiotics but not requiring surgery. Having noticed that her fatigue seemed worse during the second half of her menstrual cycle, she consulted an endocrinologist, who treated her with oral contraceptives and synthetic progesterone, neither of which helped with the fatigue or PEM.

By 2008, she had finished her master’s program and started working part-time as an environmental consultant. However, her condition continued to worsen, and she developed new symptoms of dizziness, chronic light-headedness, unstable blood pressure and heart rate, sound and vibration sensitivity, nausea, and extreme thirst, in addition to the ongoing fatigue, PEM, and pain. She consulted an integrative medicine doctor, who suggested, based on her symptoms, that she might have persistent Lyme disease, even though tests for tick-borne infections were inconclusive or negative. He treated her with a four-month trial of doxycycline, which did not ameliorate symptoms. She subsequently developed a severe sinus infection in 2010 that caused a three-month-long crash and left her bedbound. She had to abandon her career and was unable to drive after this episode.

She had sleep studies in 2005, and again in 2011, that ruled out narcolepsy, sleep apnea, and periodic leg movements. No abnormalities were observed on the electrocardiogram (EKG) or electroencephalogram (EEG). The first sleep study diagnosed atypical sleep disturbances and the second a shifted sleep cycle and poor sleep efficiency. For many years she took a small dose of Ritalin (5–10 mg) during the day to obtain a few hours of ability to focus, and occasionally Ambien at night to help her stay asleep. She also tried Provigil, without benefit.

Still in search of a treatment that would help, she started working with immunologists in other parts of the country. She was officially diagnosed with ME/CFS in 2011 by an immunologist and ME/CFS expert. Immune function testing revealed low levels of some natural killer cells and an abnormal cytokine profile, along with traces of an immune response to EBV and human herpesvirus 6 (HHV-6). Tests for other common viruses were negative. She was treated with the antiviral famciclovir (Famvir) for nine months but had no improvement in her physical condition. She was treated with immunomodulators, including Nexavir and low-dose naltrexone, but they provided no benefit, and the side effects of increased dizziness and neurological symptoms were not tolerable.

She consulted a cardiologist in 2011 and was diagnosed with neurally mediated hypotension (NMH) and postural tachycardia syndrome (POTS), common autonomic problems in pwME. Treatment with fludrocortisone (Florinef) did not help. A second cardiologist confirmed the POTS diagnosis in 2015. Treatment with extra salt and midodrine helped a little. However, her overall condition continued to deteriorate, and by 2013, she was no longer able to travel, thus ending her ability to seek treatment with doctors in other parts of the country. Even for local appointments, some requirements such as early morning appointments or long waits in noisy, brightly lit rooms, made visits impossible.

In 2015, she applied for SSDI. Her application was denied at the initial and the reconsideration levels because the vocational experts provided by the Social Security Administration claimed that she was able to work part-time, ignoring the fact that she was completely bedbound. She was unable to find a lawyer to take her case but did find a vocational expert who would confirm her level of disability. Disability payments were finally approved by an administrative law judge two weeks before she died.

She spent the last four years of her life completely bedbound. She suffered immensely from severe exhaustion, body-wide muscle and joint pain, a stiff neck, unstable blood pressure and heart rate, muscle twitches and spasms, chronic lightheadedness, sound/vibration sensitivity, nausea, food intolerances, and extreme thirst, among other symptoms. She developed mold and chemical sensitivities that made it very difficult to find housing that did not exacerbate her symptoms. She was often unable to speak and only had a brief period on rare good days when she had the cognitive energy to focus. She used her limited amount of functional time to maintain her online connections to other people and to advocate for fellow ME/CFS sufferers and environmental causes.

Since she was unable to leave her bed, she tried to find a doctor who could attend to her in her home, but this proved impossible. She had a day-time caregiver for 15 hours per week, provided by a state agency, but turnover was high. The final unbearable symptom was repeatedly waking up at night and feeling like her heart had stopped and she could not breathe. After some time, her heart would start beating erratically and she would catch a breath, but the experience was terrifying. Worn out by her prolonged struggle with pain, isolation, abysmally low quality of life, and her futile search for some possibility that her condition would improve, and although she felt loved and loving, she ended her life. 

This case demonstrates the high risk of suicide for patients with a misunderstood and difficult-to-treat disease such as ME/CFS. Similar to other chronic illnesses, suicide is many times more common in pwME than in the general population [24] and occurs at a younger average age [25]. In addition to unsupportive peer and medical interactions, risk factors for suicide include the stigma associated with the disease name “chronic fatigue syndrome,” the continual presence of pain, and often significantly decreased functionality [26].

## 5. Discussion

One of the common themes in these three case studies is the difficulty pwME encounter in finding supportive doctors and in receiving an accurate diagnosis. All three consulted tens of doctors across many specialties, and all three reported at least some interactions with dismissive or hostile health care providers. All three had the experience of being told by a doctor that they were perfectly healthy, often based on normal laboratory results, despite debilitating symptoms. The length of time to receive a diagnosis of ME/CFS was 3 years, 13 years, and 12 years, respectively. This theme highlights the need for better education of health care providers about ME/CFS. Early diagnosis and support can help pwME understand how to manage their disease through pacing and symptom management and to avoid activities, such as strenuous exercise, which exacerbate symptoms. In addition, treatment of symptoms and comorbidities can greatly improve quality of life. Recent developments, such as guidelines for treatment from the US Clinician’s Coalition [2], updated guidelines from the UK National Institute of Health and Care Excellence (NICE) [27], and updated information on the US Center for Disease Control and Prevention (CDC) website [28] are encouraging, but much more is needed.

A second theme is the difficulty imposed on pwME by the lack of a diagnostic test for ME/CFS and the lack of treatments. These deficiencies are directly related to the extremely low level of research funding for ME/CFS in the US [29] and worldwide [30]. In the US, ME/CFS research is underfunded by about a factor of 14 relative to its burden of disease [31,32]. One reason for this may be that pwME are predominantly female, and, as recently demonstrated in Mirin (2020) [33], female-dominated diseases tend to be underfunded relative to male-dominated diseases. A second reason may be that the longstanding pattern of stigmatization and psychologization of ME/CFS has discouraged funding agencies from allocating resources or positively reviewing research proposals. High profile reports, e.g., from the National Academy of Medicine in 2015, have emphasized that ME/CFS is biological in origin and that research is desperately needed [1]. The National Institutes of Health (NIH) has doubled annual funding, created three collaborative research centers, and started an intramural research project [34], but much more is needed.

A third theme is the general lack of support for pwME who are homebound or bedbound due to the fragmented and ineffective social support networks in the US. Despite the fact that most pwME cannot work full-time, or even part-time, disability support is very difficult to obtain. Applications to the US Social Security Disability Insurance program are routinely denied at the initial level and at the reconsideration level, as experienced by both Case B and Case C. In 2017, only 13,000 people received disability payments from Social Security for ME/CFS, out of potentially hundreds of thousands needing assistance [35], leading to severe financial stress and often homelessness. Medical care at home is very limited in the US, as experienced by Case C, meaning that many pwME receive no medical care at all. Better models for caring for this community exist (e.g., Kingdon et al. (2020) [36]) but would require a serious commitment from the US government in funding and resources. However, the dramatic increase in the availability of telemedicine during the COVID-19 pandemic has improved access and will hopefully remain in place.

## 6. Conclusions

We have presented three cases of people with moderate to very severe ME/CFS, demonstrating the debilitating nature of the disease and the difficulty in obtaining diagnosis and treatment. ME/CFS is not rare and the number of cases is expected to increase dramatically over the next few years due to the long-term consequences of the COVID-19 pandemic. Colloquially termed the COVID-19 long haulers, a large fraction of survivors exhibit ongoing symptoms that closely resemble ME/CFS, including fatigue, PEM, orthostatic intolerance, and cognitive difficulties [14,37]. We owe it to pwME and the COVID-19 long haulers to invest in the research to discover causes, treatments, and hopefully a cure, and to develop the medical and social support networks to improve their lives.

## Data Availability

No research data was collected.
